# Salivary Secretory Immunoglobulin A as a Candidate Biomarker of Oral Mucosal Vulnerability in Paediatric Acute Lymphoblastic Leukaemia: A Prospective Pilot Cohort Study

**DOI:** 10.3390/children13070942

**Published:** 2026-07-17

**Authors:** Ionut-Vlad Serbanica, Andreea Nicoleta Șerbănică, Karina-Doris Vihta, Andra-Daniela Marcu, Ana-Maria Bica, Cristina-Georgiana Jercan, Letitia Elena Radu, Radu Obrisca, Irina Avrămescu, Elisa Busescu, Delia Codruta Popa, Cerasela Jardan, Dima Jardan, Anca Coliță

**Affiliations:** 1 Faculty of General Medicine, “Carol Davila” University of Medicine and Pharmacy, 020022 Bucharest, Romania; ionut-vlad.serbanica@drd.umfcd.ro (I.-V.S.); andreea.serbanica@umfcd.ro (A.N.Ș.); andra.marcu@umfcd.ro (A.-D.M.); ana-maria.birsan@drd.umfcd.ro (A.-M.B.); cristina.jercan@umfcd.ro (C.-G.J.); letitia.radu@umfcd.ro (L.E.R.); radu.obrisca@drd.umfcd.ro (R.O.); irina.avramescu@drd.umfcd.ro (I.A.); elisa-maria.busescu@drd.umfcd.ro (E.B.); delia.popa@umfcd.ro (D.C.P.); cerasela.jardan@umfcd.ro (C.J.); anca.colita@umfcd.ro (A.C.); 2 Department of Pediatrics, Fundeni Clinical Institute, 022328 Bucharest, Romania; 3 Molecular Biology Laboratory, MedLife, 010719 Bucharest, Romania; djardan@medlife.ro; 4 Laboratory, Fundeni Clinical Institute, 022328 Bucharest, Romania

**Keywords:** acute lymphoblastic leukaemia, paediatric oncology, salivary sIgA, oral mucositis, dento-maxillary complications, mucosal immunity, pilot study

## Abstract

**Highlights:**

**What are the main findings?**

- Salivary secretory immunoglobulin A (sIgA) showed a non-significant decline from diagnosis to completion of intravenous chemotherapy (median 174.5, IQR 127.2–213.8 versus median 111.5, IQR 33.2–142.7, respectively; Wilcoxon signed-rank *p* = 0.084).
- A non-significant, weak inverse association was observed between baseline salivary sIgA levels and the severity of oral mucositis (Spearman ρ = −0.35, *p* = 0.20, *n* = 15).

**What is the implication of the main finding?**

- These hypothesis-generating findings require confirmation in adequately powered, prospectively designed studies before baseline salivary sIgA can be considered a candidate biomarker of mucositis risk.

**Abstract:**

**Background:** Acute lymphoblastic leukaemia (ALL) is the most common paediatric haematological malignancy. Treatment-related immunosuppression compromises mucosal integrity and contributes to oral and dento-maxillary complications, which remain a clinically relevant but insufficiently characterised source of morbidity. Salivary secretory immunoglobulin A (sIgA), the principal antibody of mucosal surfaces, may provide a non-invasive surrogate measure of local immune competence; however, its longitudinal behaviour and relationship with oral morbidity in paediatric ALL remain incompletely characterised. **Methods:** This prospective, single-centre pilot cohort study included 21 children with newly diagnosed ALL treated at the Fundeni Clinical Institute, Bucharest (March–November 2024). Salivary sIgA was measured at diagnosis (TP1) and after completion of intravenous chemotherapy (TP3). Oral mucositis (OM) was clinically assessed and graded according to WHO criteria at induction (TP2), as well as the predominant WHO grade across the post-induction chemotherapy course. Stomatological evaluation included DMFT/dmft and PUFA/pufa indices, and microbiological screening was performed at predefined time points. Statistical analyses were exploratory and included non-parametric tests and correlation analyses. **Results:** All patients developed OM at induction (TP2), with 38% experiencing severe forms (WHO grades 3–4). Baseline salivary sIgA (TP1) (*n* = 15) showed a non-significant weak inverse association with mucositis severity (Spearman ρ = −0.35, *p* = 0.20); differences across severity groups did not reach statistical significance (Kruskal–Wallis *p* = 0.43). When grouped, lower median salivary sIgA levels were observed in patients with severe mucositis compared with mild-to-moderate forms (median 91.0 mg/L (IQR 65.0–123.0) vs. 170.5 mg/L (IQR 125.0–231.8); non-significant *p* = 0.13). A non-significant decline in salivary sIgA was observed from TP1 to TP3 (median 174.5, IQR 127.2–213.8 versus median 111.5, IQR 33.2–142.7; Wilcoxon signed-rank *p* = 0.084; *n* = 10 paired measurements). Pre-existing dento-maxillary morbidity was substantial (median DMFT 4.0 (IQR 2.5–5.5), PUFA 3.0 (IQR 1.5–4); dmft 4.0 (IQR 3.0–5.0), pufa 3.0 (IQR 2.0–3.5)), but showed no statistically significant association with either mucositis grade or salivary sIgA levels. No clinically meaningful correlation was observed between salivary and intestinal sIgA levels (Spearman ρ = 0.33 overall; ρ = 0.16 after exclusion of an extreme outlier; *p* > 0.05 in both analyses), consistent with immunological compartmental independence. **Conclusions:** In this exploratory pilot cohort, no statistically significant associations were observed between salivary sIgA and oral mucositis severity or dento-maxillary indices. Although trends toward lower baseline salivary sIgA in severe mucositis and declining sIgA during induction therapy were observed, larger adequately powered studies are needed to determine whether these findings represent true biological associations.

## 1. Introduction

### 1.1. Background

Acute leukaemia (AL) accounts for approximately 40% of childhood malignancies [1]. Acute lymphoblastic leukaemia (ALL) is the most common subtype, representing around three-quarters of newly diagnosed paediatric AL cases [1] and 75–80% of childhood leukaemias overall [2]. Its incidence in the United States is estimated at 3–4 cases per 100,000 children under 15 years of age [1]. Reported peak incidence varies across studies, from 1–4 years [3] to 2–5 years [2]. Over recent decades, therapeutic advances have raised 5-year overall survival rates above 80% [1], reaching over 90% in contemporary paediatric ALL studies [4]. Consequently, supportive and complementary care has become an essential component of comprehensive management in these patients [1,2,3,4]. The chemotherapy regimens required to achieve and consolidate remission in ALL exert a profound negative impact on the integrity of the oral mucosa, salivary secretory function, oral microbiome composition and local immunological parameters, with direct repercussions throughout the entire dento-maxillary system [5,6]. These treatment-related complications are not merely symptomatic inconveniences—they represent major sources of morbidity, impair nutritional status, facilitate opportunistic infections, compromise treatment compliance and adversely affect quality of life [6,7].

### 1.2. Oral and Dento-Maxillary Complications in Paediatric ALL

Oral mucositis, defined as inflammatory and ulcerative lesions of the oral mucosa secondary to cytotoxic therapy, represents one of the most frequent and distressing complications of induction chemotherapy, occurring in 60–80% of paediatric ALL patients [6,7,8,9]. Severe forms (WHO grades 3–4) are characterised by painful, sometimes debilitating, confluent, deeply ulcerative lesions that impair swallowing, speech and oral intake, exert a significant nutritional and psychological burden, promote systemic opportunistic infections and may necessitate treatment interruptions, thereby adversely affecting morbidity and quality of life [5,8,10,11]. The pathobiology of mucositis encompasses direct cytotoxic injury to basal epithelial cells, activation of pro-inflammatory nuclear factor-κB (NF-κB) signalling pathways, and secondary microbial colonisation, culminating in mucosal ulceration and impaired epithelial barrier function [7,10].

At the level of dental structures, chemotherapy induces significant alterations, including enamel hypoplasia, enamel demineralisation, root morphology anomalies, delayed tooth eruption, and increased susceptibility to dental caries and periodontal disease [12,13,14,15]. These changes represent measurable outcomes of treatment-related toxicity, reliably quantified using validated clinical indices—the DMFT (decayed, missing, filled teeth) index for caries burden assessment and the PUFA index (pulp involvement, ulceration, fistula, abscess) for the severity of untreated odontogenic infections [12,16,17,18,19,20].

### 1.3. Salivary Secretory IgA and Mucosal Immunity

Secretory immunoglobulin A (sIgA) is the predominant antibody isotype at mucosal surfaces, where it plays a central and non-redundant role in maintaining immunological homeostasis and providing protection against pathogens through mechanisms of local humoral immunity [21,22]. In its dimeric form, stabilised by the J chain and the secretory component, sIgA is released into saliva, tears, intestinal secretions and other mucosal fluids. The secretory component confers resistance to proteolytic degradation, ensuring structural stability within the hostile environment of mucosal cavities. At these surfaces, sIgA exerts immune exclusion through the neutralisation of pathogens, agglutination of commensal bacteria, prevention of epithelial adhesion and modulation of local inflammatory responses—without activating complement or triggering systemic inflammation [21,22].

Within the oral cavity, sIgA represents the first immunological line of defence against cariogenic bacteria—including *Streptococcus mutans* and related viridans streptococci (*S. oralis*, *S. mitis*, *S. sanguinis*) with comparable acidogenic and biofilm-forming capacities—fungal colonisation (*Candida* spp.) and viral pathogens [23,24]. A deficit of salivary sIgA—whether constitutive or induced by chemotherapy-mediated immunosuppression—favours pathological microbial proliferation, increases epithelial vulnerability and predisposes to the full spectrum of oral and dento-maxillary complications observed during ALL treatment [21,22].

Importantly, mucosal IgA production is largely independent of systemic humoral immunity—the majority of IgA-secreting plasma cells reside within mucosa-associated lymphoid tissues (MALT, GALT) and operate through distinct regulatory mechanisms [21,22]. This immunological autonomy implies that serum IgA measurement cannot reliably reflect local mucosal immune competence, and that dedicated salivary sIgA assessment is required for meaningful clinical evaluation in paediatric oncology [22,23,24,25,26,27].

In this study, we aimed to investigate the role of salivary sIgA in paediatric acute lymphoblastic leukaemia, assessing its longitudinal changes, relationship with OM and dento-maxillary complications, and correlation with intestinal and serum IgA. This was an exploratory, hypothesis-generating pilot study with salivary sIgA as the prespecified exploratory focus; the remaining oral, dento-maxillary and microbiological parameters were assessed as exploratory variables, with no single prespecified primary outcome.

## 2. Materials and Methods

### 2.1. Study Design

A prospective, single-centre, observational pilot cohort study was conducted at the Paediatric Haematology–Oncology Clinic of the Fundeni Clinical Institute, Bucharest, Romania, between March and November 2024. The primary focus was the longitudinal evaluation of oral and dento-maxillary complications in paediatric patients with ALL, integrated with serial sIgA as a candidate non-invasive mucosal immune biomarker. The study was reported in accordance with the STROBE guidelines for observational cohort studies [28]. As this was a non-interventional observational study, prospective registration in a clinical trial registry was not required. Treatment followed standard paediatric oncology protocols, and the study was observational with respect to that care; the orthopantomogram and saliva collection were research evaluations added to routine clinical care, performed under institutional ethics approval (protocol 12504, 12 March 2024) with informed consent, and did not influence treatment decisions.

#### 2.1.1. Inclusion Criteria

- Paediatric patients with a confirmed de novo diagnosis of ALL at the Fundeni Clinical Institute;
- Written informed consent obtained from parents or legal guardians prior to any study procedure.

#### 2.1.2. Exclusion Criteria

- Administration of intravenous immunoglobulins within 30 days preceding enrolment;
- Confirmed history of autoimmune disease or primary immunodeficiency;
- Pre-existing severe acute oral infection unrelated to ALL diagnosis.

### 2.2. Diagnosis of ALL and Risk Group Classification

ALL diagnosis was established according to morphological (FAB classification), immunophenotypic (flow cytometry) and cytogenetic criteria, supplemented by conventional karyotype, FISH, NGS and MLPA for comprehensive molecular characterisation.

Risk stratification was performed in accordance with current European paediatric oncology guidelines, incorporating leukaemia immunophenotype (B-cell precursor vs. T-cell ALL), cytogenetic and molecular genetic findings, initial leucocyte count, CNS involvement, and early treatment response, assessed by the peripheral blood blast count on day 8 and by minimal residual disease (MRD) evaluation at days 15, 33 and 78. Standard-risk group (SRG) comprised patients with B-cell precursor ALL, favourable cytogenetics (*ETV6-RUNX1* fusion or high hyperdiploidy), absence of CNS involvement, and adequate MRD clearance at defined induction time points. The high-risk group (HRG) included patients with T-cell ALL (including ETP-ALL), adverse cytogenetic abnormalities (*t(9;22)/BCR-ABL1*, *t(4;11)/KMT2A-AFF1*, *iAMP21*), unfavourable somatic mutational profile, elevated initial leucocyte count, CNS involvement, or insufficient MRD response at defined time points [5,29,30].

Treatment was administered according to a four-phase chemotherapy protocol (induction, consolidation, re-induction and maintenance) in accordance with current European paediatric oncology recommendations [31]. Patients were treated based on the AIEOP-BFM ALL protocol, with risk-group stratification as defined therein. Minimal residual disease was assessed by multiparametric flow cytometry and molecular PCR at the time points indicated by the protocol.

### 2.3. Oral and Dento-Maxillary Assessment

Each participant underwent a comprehensive oral and dento-maxillary evaluation performed by an experienced specialist in dentistry, using artificial light, a dental mirror and a calibrated periodontal probe. The following clinical parameters were assessed:

OM: presence, distribution and severity graded according to the World Health Organization (WHO) Five-grade Oral Mucositis Grading Scale (grades 0–4) [31] by the medical team involved in patient care, with grading independently performed by at least two observers at each examination. At least one assessor was blinded to the salivary sIgA results. All patients were evaluated at TP2, during the induction phase, between the second and third chemotherapy administrations, and were subsequently reassessed throughout the treatment course whenever oral mucositis was clinically evident, with the observed mucositis grade recorded for the entire treatment period. Grades 0–2 were classified as absent or mild-to-moderate mucositis; grades 3–4 as severe mucositis requiring nutritional modification or support. Main analyses focused on OM at induction (TP2), corresponding to the expected peak of toxicity during induction chemotherapy. Sensitivity analysis also considered the predominant mucositis WHO grade across the post-induction chemotherapy courses.

Dental caries status: quantified using the DMFT (permanent teeth: decayed, missing, filled)/dmft (primary teeth: decayed, missing, filled) index [18].

Odontogenic infection burden: assessed using the PUFA/pufa index (P/p = pulp involvement; U/u = ulceration of the oral mucosa; F/f = fistula; A/a = abscess) [17]. A PUFA score ≥ 4 was considered indicative of severe odontogenic infection burden.

Periodontal status: gingival index, presence of spontaneous gingival bleeding and bleeding on probing.

Additional oral findings: xerostomia, bacterial plaque accumulation, dental mobility, papillary atrophy, candidiasis, oral petechiae and dental enamel hypoplasia.

Panoramic dental radiography: orthopantomogram performed to identify delayed tooth eruption, widening of the periodontal ligament space, root dysmorphology and diffuse radiolucent areas.

The following assessments were performed at predefined study timepoints. TP1 corresponded to baseline evaluation and included salivary sample collection for sIgA assessment. TP2 represented the peak phase of induction chemotherapy and was used exclusively for OM evaluation; no saliva samples were collected at this timepoint. OM severity was assessed according to the WHO grading scale (grades 0–4) at every clinical visit during which oral mucosal lesions were identified throughout the chemotherapy period. TP3 corresponded to completion of intravenous chemotherapy and initiation of maintenance therapy and included repeated salivary sIgA assessment, comprehensive oral examination with DMFT/dmft and PUFA/pufa indices, as well as panoramic dental radiography (OPG) and GI-MAP assay, which included microbial and intestinal sIgA determination. Saliva was not collected at TP2 due to financial and logistical constraints. Consequently, the temporal relationship between changes in salivary sIgA levels and the peak severity of mucositis could not be established, limiting the ability to draw causal inferences. This issue is acknowledged and discussed in the Limitations section.

### 2.4. Saliva Collection and sIgA Determination

Unstimulated whole saliva samples were collected in accordance with a standardised protocol to minimise biological variability. Samples were obtained between 08:00 and 10:00 h, prior to oral hygiene procedures or any food or fluid intake, from hospitalised paediatric patients within 24 h of confirmed ALL diagnosis and prior to initiation of chemotherapy (TP1), and at completion of intravenous chemotherapy (TP3).

Saliva was collected non-invasively by passive drooling into a sterile container over 5 min, with a minimum required volume of 2 mL. Samples were centrifuged at 3000 rpm for 10 min at 4 °C; the supernatant was aliquoted and stored at −80 °C until analysis. Salivary secretory IgA (sIgA) was quantified at an accredited reference laboratory (MVZ Labor Dr. Limbach & Kollegen, Heidelberg, Germany; reference range 20–200 mg/L). Saliva samples were collected and processed at Bioclinica (Bucharest, Romania) and shipped under standardised conditions prior to analysis. Salivary IgA concentrations were measured by nephelometry using the N Latex IgA reagent (REF: OQAI11; Siemens Healthineers, Marburg, Germany) on a BN II analyser (Siemens Healthineers, Germany). All samples yielded quantifiable sIgA concentrations. The reference range provided by the laboratory (20–200 mg/L) is a generic interval, not age-stratified for the paediatric salivary matrix; it was therefore used for descriptive purposes only and was not applied as a normative threshold in the statistical analyses.

### 2.5. Oro-Pharyngeal Microbiological Assessment

Nasal and pharyngeal swabs were collected at diagnosis under fasting conditions and prior to initiation of chemotherapy, and subsequently prior to each major chemotherapy phase (induction, consolidation and re-induction), for serial assessment of oropharyngeal dysbiosis. Microbiological analysis focused on fungal screening—particularly *Candida albicans*—and bacterial identification, with emphasis on Streptococcus group species due to their cariogenic and opportunistic pathogenic potential.

### 2.6. Intestinal Microbiota Analysis

Intestinal microbiota assessment was performed at diagnosis (TP1), prior to initiation of ALL treatment, and repeated upon completion of intravenous chemotherapy (TP3). Stool samples were collected under sterile conditions in accordance with protocol requirements. Microbiota analysis was conducted using the GI Microbial Assay Plus (GI-MAP; Diagnostic Solutions Laboratory, Alpharetta, GA, USA), a quantitative polymerase chain reaction (qPCR)-based clinical assay designed to detect and quantify a standardised panel of gastrointestinal microorganisms, including commensal bacteria, opportunistic pathogens, fungi, viruses, and parasites. The assay was employed to identify selected intestinal microbial taxa with potential pro-inflammatory and indirect cariogenic relevance to the dento-maxillary system. In addition to microbial profiling, the GI-MAP assay includes assessment of intestinal immunological markers, such as sIgA, which may provide additional information regarding mucosal immune function and intestinal immune response. Accordingly, intestinal sIgA levels were evaluated using the GI-MAP assay at both TP1, in all enrolled patients, and TP3, in only the first ten patients who completed intravenous chemotherapy, as repeated testing was limited by the high cost of the assay. GI-MAP was selected because it was the only molecular gut microbiota assay clinically available to the unit, as no alternative method was reimbursed by the national health system; the assay cost was covered by the association of parents of children with oncological diseases. It constitutes a standardised and validated qPCR panel with a clinically practical turnaround time, in contrast to 16S rRNA sequencing or shotgun metagenomics; we acknowledge that it does not provide species-level resolution (e.g., *S. mutans* vs. *S. salivarius*) or comprehensive microbiome characterisation.

Unlike broad-spectrum microbiome profiling techniques, such as 16S rRNA gene sequencing or shotgun metagenomic analysis, the GI-MAP assay represents a targeted molecular approach and does not provide comprehensive characterisation of the intestinal microbiome. Consequently, interpretation of the present findings was limited to the specific taxa included in the assay panel, and non-detection of individual microorganisms should not be interpreted as evidence of their complete absence from the intestinal microbial community.

### 2.7. Ethical Considerations

All procedures were conducted in accordance with the Declaration of Helsinki [32] and received approval from the institutional ethics committee of the Fundeni Clinical Institute. Written informed consent was obtained from the parents or legal guardians of all paediatric participants prior to enrolment. Given the observational nature of the study, all diagnostic and therapeutic interventions were performed according to standard paediatric oncology protocols and were not influenced by salivary sIgA results or any other study procedure.

Artificial intelligence-based language tools (Claude 5 Anthropic; ChatGPT, GPT-5.5, OpenAI, San Francisco, CA, USA) were used exclusively to support English language editing, translation, and improvement of manuscript clarity and readability. All study design, patient recruitment, data collection, statistical analyses, figure generation, scientific interpretation and conclusions were performed solely by the authors. The AI tools were not involved in any aspect of the research process. The authors assume full responsibility for the integrity, accuracy and originality of the reported work.

### 2.8. Statistical Analysis

Statistical analyses were performed using R software, version 4.5.2 (2025-10-31) (R Core Team, Vienna, Austria). We used the stat_compare_means function from the ggpubr package to add mean comparison *p*-values to boxplots created with the ggplot function from ggplot2. For comparisons between two groups, Wilcoxon tests were used, and for three or more groups, Kruskal–Wallis tests were applied. The correlation between continuous measurements was evaluated using Spearman correlations. Spearman’s rank-order correlation was used to assess the association between dental indices (dmft, DMFT, pufa, PUFA) and clinical/salivary outcomes (salivary sIgA TP1, OM TP2, salivary sIgA TP3). This non-parametric method was chosen due to the small sample size and non-normal distribution of variables. Analyses were conducted using complete-case data (i.e., observations with missing values were excluded pairwise). For each correlation, the Spearman correlation coefficient (ρ), *p*-value, and number of complete observations (n) were reported.

To account for multiple testing, *p*-values were adjusted using the Benjamini–Hochberg false discovery rate (FDR) procedure. This was a convenience sample; no a priori sample size or statistical power calculation was performed, and no primary outcome was pre-specified. All analyses were therefore exploratory and hypothesis-generating, and none were confirmatory.

## 3. Results

### 3.1. Patient Cohort Characteristics

The study cohort comprised 21 paediatric patients diagnosed with ALL, with a median age of 8 years (IQR 4–12; range 2–17 years). At the time of analysis (January 2026), one patient had died; five patients were censored following transfer to other centres for continuation of treatment after induction, and 15 patients remained in active follow-up. Fourteen patients (67%) were male and seven (33%) were female. Eleven patients (52%) originated from urban areas, eight (38%) from rural areas, and two (10%) from the Republic of Moldova. Sixteen patients (76%) were diagnosed with B-cell ALL and five (24%) with T-cell ALL. According to risk stratification, six patients (29%) were classified as standard risk (SRG), eleven (52%) as intermediate risk and four (19%) as high risk (Table 1).

### 3.2. Oral Mucositis Severity and Association with Baseline Salivary sIgA

#### 3.2.1. Salivary sIgA

At diagnosis (TP1), salivary sIgA measurements were available for 15/21 patients (71%). Samples could not be obtained at TP1 from 6/21 patients (29%), all aged under 4 years (range 2–4 years), due to insufficient saliva volume; these patients provided adequate samples at TP3. The median salivary sIgA concentration at diagnosis was 140 mg/L (IQR 101.5, 182, Table 2). As no healthy paediatric control group was included, absolute salivary sIgA values could not be interpreted against normative references. Analyses were therefore restricted to within-patient longitudinal changes and between-group comparisons.

**Figure 1:**
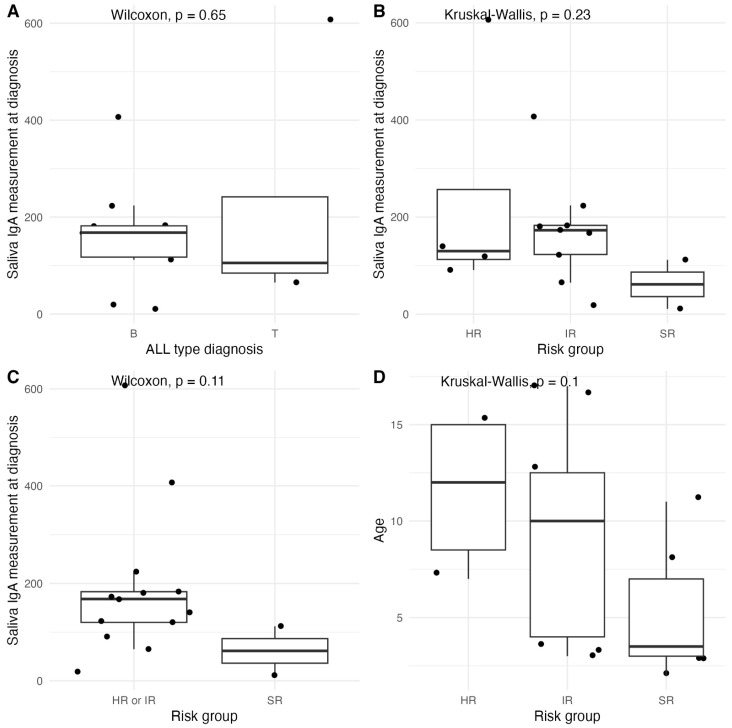
Salivary sIgA levels at diagnosis, stratified by ALL subtype (**A**) and risk groups (**B**), grouped risk groups (**C**), as well as age distribution by risk group (**D**).

No statistically significant differences in baseline salivary sIgA were observed according to ALL immunophenotype (B median 168.0, IQR 117.5–182.0 vs. T median 105.5, IQR 84.5–241.8, Wilcoxon test *p* = 0.66) or risk group (HR median 130.0, IQR 112.8–256.8, IR median 173.0, IQR 123.0–183.0, SR median 61.5, IQR 36.25–86.75, Kruskal–Wallis test *p* = 0.23) (Figure 1A,B). When intermediate- and high-risk patients were pooled and compared with the standard-risk group, lower salivary sIgA values were observed in the standard-risk group (HR or IR median 168.0, IQR 120.0–183.0 vs. SR median 61.5, IQR 36.25–86.75, Wilcoxon test *p* = 0.11, Figure 1C), likely reflecting both age-related physiological differences—given the younger, although not statistically significant, median age of standard-risk patients (HR median 12.0, IQR 8.5–15, IR median 10.0, IQR 4.0–12.5, SR median 3.5, IQR 3.0–7.0, Kruskal–Wallis *p* = 0.1, Figure 1D)—and the lower intensity of the chemotherapy regimens administered within this stratum.

#### 3.2.2. Oral Mucositis During Chemotherapy

The distribution of OM severity at induction (TP2) across ALL subtypes and risk groups is presented in Table 3. Grade 2 mucositis was the most frequently observed, affecting 9 of 21 patients (42.9%), while clinically significant lesions—defined as Grade 3 or 4—occurred in 8 patients (38.1%). The single case of Grade 4 mucositis was recorded in a T-cell ALL patient classified as high-risk. Within the intermediate-risk group, Grades 2 and 3 predominated (5 patients each), likely reflecting the intensity of the chemotherapy regimens administered within this stratum.

#### 3.2.3. Association Between Baseline Salivary sIgA and Oral Mucositis Severity

**Figure 2:**
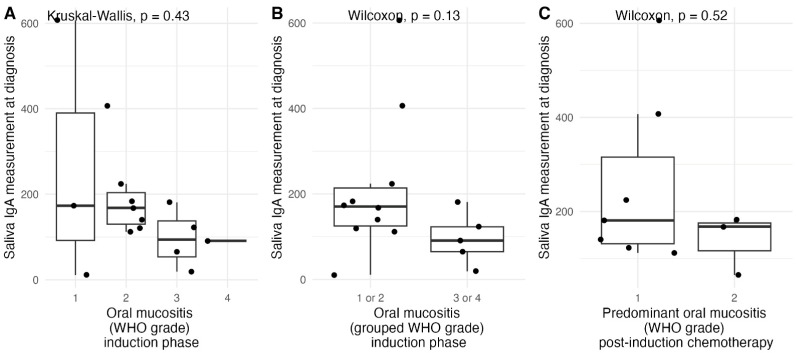
Boxplots of salivary sIgA levels at diagnosis, stratified by OM WHO grade during the induction phase (TP2) (**A**), OM WHO grade during the induction phase (TP2) grouped as grades 1–2 and 3–4 (**B**), and by the predominant OM WHO grade across the post-induction chemotherapy course (**C**). Note: Paired data were available for 15/21 (71%) patients in (**A**,**B**), and for 10/21 (48%) patients in (**C**).

Salivary sIgA at diagnosis demonstrated a weak inverse correlation with OM severity, although this did not reach statistical significance (Spearman ρ = −0.35, *p* = 0.20, *n* = 15). When OM (TP2) grades were compared as four independent groups, salivary sIgA concentrations did not differ significantly between grades (grade 1: median 173.0 mg/L, IQR 92.0–390.0; grade 2: median 168.0 mg/L, IQR 130.0–203.5; grade 3: median 94.0 mg/L, IQR 53.5–137.5; grade 4: one patient, 91.0 mg/L; Kruskal–Wallis test, *p* = 0.43; Figure 2A). When OM grades were grouped as mild-to-moderate (grades 1–2) and severe (grades 3–4), median salivary sIgA was lower in patients who developed severe mucositis (91.0 mg/L, IQR 65.0–123.0) than in those with mild-to-moderate mucositis (170.5 mg/L, IQR 125.0–231.8), although the difference was not statistically significant (Wilcoxon rank-sum test, *p* = 0.13; Figure 2B). Similarly, baseline salivary sIgA appeared to be lower in patients whose predominant OM severity across the post-induction chemotherapy course was grade 2 compared with grade 1, but this difference was not statistically significant (grade 1: median 181.0 mg/L, IQR 131.5–315.5; grade 2: median 168.0 mg/L, IQR 116.5–175.5; *p* = 0.52; Figure 2C).

### 3.3. Longitudinal Changes in Salivary sIgA (TP1 vs. TP3)

**Figure 3:**
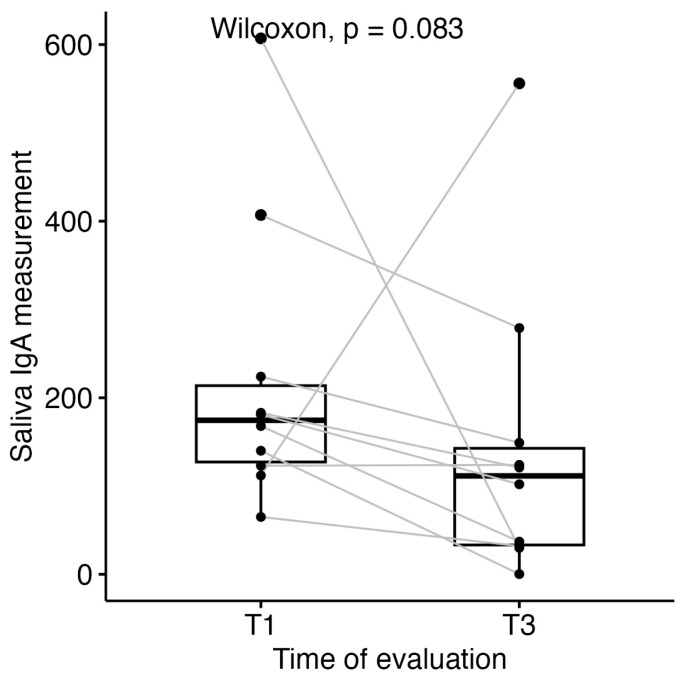
Boxplots of salivary sIgA levels at diagnosis (TP1) and at the completion of chemotherapy (TP3).

Paired measurements were available for 10 of 21 patients (48%). Among these patients, median salivary sIgA decreased from 174.5 mg/L (IQR 127.2–213.8) at diagnosis to 111.5 mg/L (IQR 33.2–142.7) following chemotherapy. This reduction did not reach statistical significance (Wilcoxon signed-rank test, *p* = 0.083, Figure 3).

### 3.4. Dento-Maxillary Morbidity Burden and Its Relationship with Salivary sIgA and Mucositis Severity

We found that 10/21 (48%) patients younger than 8 years old only presented primary teeth, 6/21 (29%) between 8 and 11 years old inclusive presented mixed dentition, while the remaining 5/21 (24%) patients aged 12 and above presented permanent dentition alone. Median DMFT was 4.0 (IQR 2.5–5.5) and median PUFA was 3.0 (IQR 1.5–4.0); in the primary dentition stratum, median dmft was 4.0 (IQR 3.0–5.0) and median pufa was 3.0 (IQR 2.0–3.5).

Spearman correlation analysis revealed generally weak to moderate associations between dental indices and the evaluated outcomes (Table 4). Most correlations were not statistically significant.

Before adjustment for multiple comparisons, strong negative correlations were observed between DMFT and salivary sIgA TP1 (ρ = −0.83, *p* = 0.021, *n* = 7) and between PUFA and salivary sIgA TP3 (ρ = −0.81, *p* = 0.027, *n* = 7). However, after applying the Benjamini–Hochberg correction, these associations were no longer statistically significant (adjusted *p* = 0.162 for both). The strong correlations observed in small subsamples (*n* = *7*) may reflect instability and should be interpreted cautiously.

All other correlations were weak to moderate in magnitude (|ρ| ≤ 0.55) and non-significant (adjusted *p* > 0.05). No meaningful associations were observed between dental indices and OM.

Overall, the findings do not provide evidence of statistically significant monotonic relationships between the investigated variables. Results should be interpreted with caution due to the small number of complete observations in some analyses.

**Figure 4:**
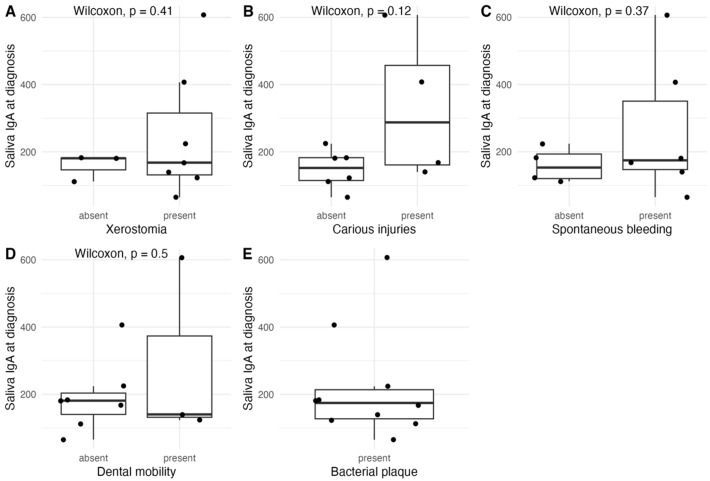
Comparison of salivary sIgA levels at diagnosis in those with vs. without oral cavity changes identified on stomatological examination. (**A**) xerostomia; (**B**) carious injuries; (**C**) spontaneous bleeding; (**D**) dental mobility; (**E**) bacterial plaque.

Salivary sIgA levels at diagnosis were compared according to oral health conditions using the Wilcoxon rank-sum test. No statistically significant differences were observed between groups for any of the evaluated conditions. Specifically, salivary sIgA levels did not differ between patients with and without xerostomia (*p* = 0.41), carious injuries (*p* = 0.12), spontaneous bleeding (*p* = 0.37), or dental mobility (*p* = 0.50). All patients presented bacterial plaque. Although some variability and higher median values were noted in participants with certain conditions (e.g., carious injuries and spontaneous bleeding), these differences did not reach statistical significance (Figure 4).

**Figure 5:**
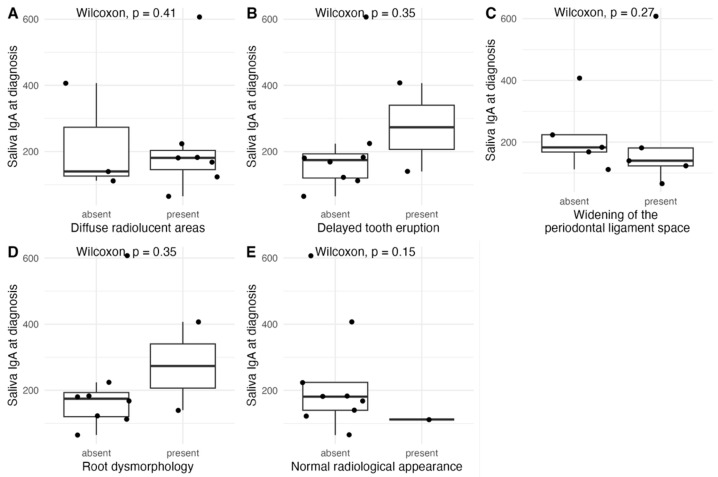
Distribution of salivary sIgA levels at diagnosis in those with vs. without dento-maxillary radiological findings on orthopantomography. (**A**) diffuse radiolucent areas; (**B**) delayed tooth eruption; (**C**) widening of periodontal ligament space; (**D**) root dysmorphology; (**E**) normal radiological appearance.

Salivary IgA levels at diagnosis did not differ significantly according to any of the evaluated radiographic findings (Figure 5). Specifically, no significant differences were observed for diffuse radiolucent areas (Wilcoxon, *p* = 0.41), delayed tooth eruption (*p* = 0.35), widening of the periodontal ligament space (*p* = 0.27), root dysmorphology (*p* = 0.35), or normal radiological appearance (*p* = 0.15). Although higher median salivary IgA levels were observed in patients with delayed tooth eruption and root dysmorphology versus those without, these trends were not statistically significant.

### 3.5. Oro-Pharyngeal and Intestinal Microbiological Profile

Nasal and pharyngeal swabs were obtained prior to each major chemotherapy phase. Microbiological screening results and infectious complications are summarised in Table 5. Opportunistic pathogens identified included *Candida* spp., *Streptococcus* spp., *Enterococcus* spp., *Staphylococcus aureus* and multidrug-resistant organisms. Episodes of bacteraemia with a presumed oral source were documented.

**Figure 6:**
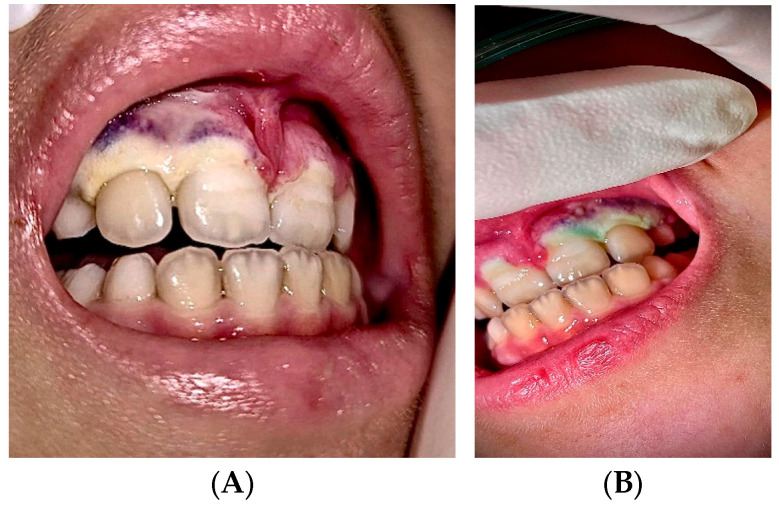
Ulceronecrotic gingivitis involving teeth 2.1–2.3 (images from the personal archive Fundeni Clinical Institute). (**A**) Frontal clinical view showing gingival ulceronecrotic lesions covered by a grayish-white pseudomembrane; (**B**) Lateral clinical view of the same lesion, with retraction of the cheek/lip, showing the extent of the pseudomembranous coating across the gingival surface.

A notable case involved a 7-year-old female with very high-risk ETP-ALL presenting a baseline salivary sIgA of 91 mg/L, in whom pharyngeal culture identified *Stenotrophomonas maltophilia*. During the neutropenic phase, she developed severe ulceronecrotic gingivitis involving teeth 2.1–2.3, with extension to the alveolar bone, grade III tooth mobility and grade 4 OM (see Figure 6). While suggestive, this observation is anecdotal and does not establish causality.

**Figure 7:**
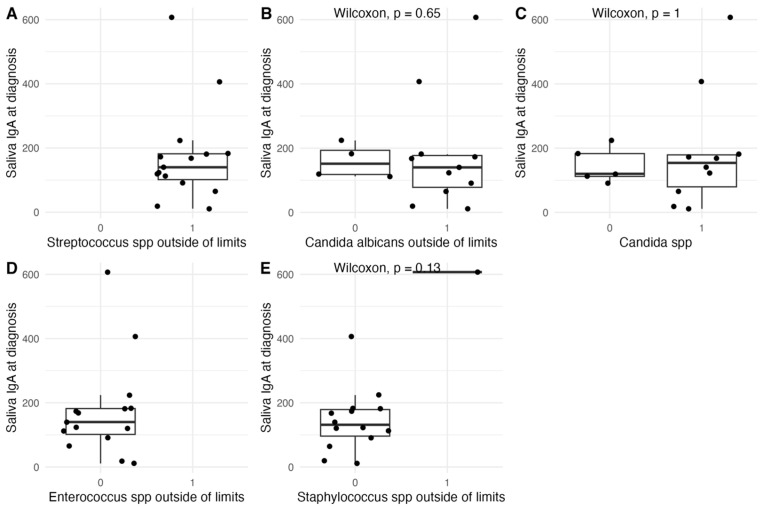
Distribution of salivary sIgA levels at diagnosis in those with vs. without *Streptococcus* spp. (**A**), *Candida albicans* (**B**), *Candida* spp. (**C**), *Enterococcus* spp. (**D**), *Staphylococcus* spp. (**E**) within (0) vs. outside (1) of the assay’s established reference range.

GI-MAP analysis identified intestinal species with indirect pro-inflammatory relevance, including *Streptococcus* spp. (above the assay’s established reference range in all patients), *Candida albicans*, *Candida* spp., *Enterococcus* spp. (within the assay’s established reference range in all patients) and *Staphylococcus* spp., without specific identification of oral cariogenic species (*S. mutans*, *S. mitis*, *S. sanguinis*). No differences in salivary sIgA levels were observed in patients with levels within versus outside of the assay’s established reference range (Figure 7).

### 3.6. Immunological Independence of Salivary Versus Intestinal sIgA

Intestinal sIgA measurements at diagnosis, by the GI-MAP assay, were available for 19/21 patients (90%), with a median of 545 mg/L (IQR 212–942). Five patients had values below 210 mg/L, while one patient exhibited an extreme value exceeding 6000 mg/L. Both salivary and intestinal sIgA data were available for 14/21 patients (67%).

No significant correlation was observed between salivary and intestinal sIgA levels (ρ = 0.33, *p* = 0.25). After exclusion of an extreme outlier, the association weakened further (ρ = 0.16, *p* = 0.60), suggesting relative independence between mucosal compartments.

### 3.7. Availability and Distribution of Serum and Salivary sIgA Data (Normal vs. Reduced Levels)

Serum sIgA data were available for 17/21 patients (81%); 12/17 (71%) had normal values and 5/17 (29%) exhibited reduced values. Actual serum sIgA levels were not available in our dataset, just the binary split. Both salivary and serum sIgA measurements were available for 12/21 patients (57%), of whom only one patient exhibited low serum sIgA at diagnosis. This patient also exhibited the lowest salivary sIgA value in the cohort.

## 4. Discussion

This prospective pilot cohort study provides integrated clinical, immunological, and microbiological data exploring whether salivary sIgA may be associated with oral mucosal vulnerability in paediatric ALL. Although no statistically significant associations were identified, several observations—including the universal occurrence of oral mucositis, a weak inverse correlation between baseline salivary sIgA and mucositis severity, lower baseline salivary sIgA in patients with severe mucositis, and a longitudinal decline in salivary sIgA during chemotherapy—were directionally consistent with a potential relationship between salivary sIgA and mucosal immune status. Given the exploratory design and limited sample size, these observations should be interpreted cautiously and require confirmation in adequately powered studies.

### 4.1. Universal Oral Morbidity and Mucositis Severity

The 100% incidence of OM observed in this cohort, with 38% severe forms (WHO grades 3–4), is consistent with prior reports highlighting the high mucotoxicity of ALL induction regimens [1,2,3]. Although reported incidence rates vary (50–80%), differences likely reflect heterogeneity in treatment protocols, grading systems, and assessment frequency [8,33]. These findings reinforce the clinical need for systematic oral monitoring and early preventive intervention in this population [11].

The universal occurrence of mucositis underscores the vulnerability of the oral mucosa to cytotoxic injury. Although no statistically significant association was identified, baseline salivary sIgA showed a weak inverse correlation with mucositis severity, with lower median concentrations observed among patients who developed severe oral mucositis. This direction of effect is consistent with current pathobiological models, in which impaired mucosal immune defence may facilitate epithelial damage and delayed healing [7,23]. However, these findings should be interpreted cautiously given the limited sample size and exploratory nature of the study. Mucositis severity is also expected to vary across treatment phases: assessment timepoints corresponded to defined phases (notably TP2 during induction), during which agents with differing mucosal toxicity were administered, in particular corticosteroids, vinca alkaloids, an anthracycline (doxorubicin) and PEG-asparaginase during induction phase IA, followed by cyclophosphamide, a purine analogue and cytarabine during induction phase IB, with subsequent risk-adapted therapy (Protocol M in the standard- and intermediate-risk groups and high-risk blocks in the high-risk group); this phase-dependent exposure is a further factor influencing mucositis severity.

### 4.2. Dento-Maxillary Morbidity Burden

The median DMFT of 4.0 (IQR 2.5, 5.5) and PUFA of 3.0 (IQR 1.5, 4) documented in this cohort reflect a substantial pre-existing dento-maxillary morbidity burden, consistent with European paediatric oncology dental studies reporting elevated caries and odontogenic infection indices in ALL patients relative to age-matched controls [16,19]. Despite their methodological advantages—reproducibility, standardisation, and low implementation cost—DMFT and PUFA indices remain underutilised in paediatric oncology clinical practice [17]. Their systematic deployment in the present study demonstrates that quantification of oral morbidity burden is feasible within an oncological setting and provides a reproducible framework for future comparative studies.

No statistically significant correlations were identified between DMFT or PUFA scores and either mucositis grade or baseline salivary sIgA levels. This absence likely reflects fundamentally different pathogenic mechanisms: acute mucositis is driven by direct cytotoxic epithelial injury, whereas dento-maxillary disease develops over months to years through cariogenic and periodontal pathways largely independent of acute mucosal events [13,20]. The limited statistical power of this pilot cohort (*n* = 21) may additionally have precluded detection of associations of small-to-moderate effect size; adequately powered prospective studies are required to clarify these relationships.

Although not statistically significant after correction, the strong negative correlations observed between DMFT/PUFA and salivary sIgA at TP3 may suggest a potential relationship that warrants further investigation. These findings should be considered hypothesis-generating and require confirmation in larger, adequately powered studies.

The non-significant association between greater dental caries burden and lower baseline salivary sIgA raises the possibility that impaired mucosal immunity may contribute to broader oral vulnerability beyond the acute mucositis episode; this remains a hypothesis warranting prospective investigation [20,21].

### 4.3. Inverse Correlation Between sIgA and Mucositis Severity

Although not statistically significant, both the inverse correlation between baseline salivary sIgA and mucositis severity and the tendency toward lower salivary sIgA values in patients who developed severe mucositis were directionally consistent with current biological understanding of mucosal immune defence. These observations require confirmation in larger, adequately powered studies. Salivary sIgA contributes to epithelial defence through multiple mechanisms: neutralisation of bacterial and viral pathogens, inhibition of streptococcal adhesion to mucosal surfaces, modulation of the local inflammatory response via non-complement-activating immune exclusion, and maintenance of the protective mucus layer [21,22]. Pre-existing depletion of this immune barrier at chemotherapy initiation plausibly facilitates deeper epithelial injury, slower re-epithelialisation and more severe ulceration in response to cytotoxic insult, a mechanistic sequence supported by both in vitro and clinical observational data [7,8]. Given the limited sample size and the absence of statistical significance, these findings should be considered preliminary and hypothesis-generating and require validation in larger, adequately powered cohorts. The relationship between salivary sIgA and mucositis severity may additionally be influenced by age, treatment intensity, risk group, infection status, oral hygiene and baseline dental pathology; these potential confounders preclude attributing mucosal vulnerability predominantly to salivary sIgA.

### 4.4. Immunological Independence of Salivary sIgA from Systemic and Intestinal Compartments

The absence of a clinically meaningful correlation between salivary sIgA and intestinal IgA levels (Spearman ρ = 0.16, *p* = 0.60, after exclusion of an extreme intestinal sIgA outlier) is consistent with the compartmental autonomy of mucosal sIgA homeostasis, as salivary and intestinal sIgA are regulated by distinct mucosal inductive sites within the MALT network, with limited cross-compartmental communication under steady-state conditions [24,25]. The single patient with low serum sIgA in this cohort also presented the lowest baseline salivary sIgA at diagnosis, an observation aligned with this immunological framework [24,26]; however, given the absence of additional cases, no systematic relationship can be inferred from this single observation.

These findings suggest that serum or intestinal sIgA measurement may not reliably reflect salivary sIgA levels when the clinical question pertains to oral mucosal immune competence. If confirmed in larger cohorts, dedicated salivary sIgA assessment may be warranted as an independent parameter in the baseline immunological evaluation of ALL patients.

### 4.5. Microbiological Landscape

The microbiological findings provide ecological context for the clinical consequences of sIgA depletion. The detection of *Candida* spp., *Streptococcus viridans* group species, *Enterococcus* spp., and *Staphylococcus aureus* in oropharyngeal and intestinal samples is consistent with the presence of opportunistic microorganisms commonly reported in immunocompromised paediatric populations receiving chemotherapy [9,34,35,36]. Given the targeted qPCR-based design of the GI-MAP assay, these findings reflect the detection of selected microorganisms included in the assay panel rather than comprehensive characterisation of intestinal microbial diversity or global microbiome composition. Consequently, the present study cannot infer broader microbiome alterations or expansion of opportunistic taxa beyond the organisms specifically assessed. The documented episodes of multidrug-resistant organism (MDRO) bacteraemia and *Clostridium difficile* colitis further illustrate the systemic clinical consequences of mucosal immune dysfunction in this population [37].

The case of a 7-year-old patient with very high-risk ETP-ALL—presenting a baseline salivary sIgA of 91 mg/L, pharyngeal colonisation with *Stenotrophomonas maltophilia,* and subsequent severe ulceronecrotic gingivitis with alveolar bone involvement—illustrates a plausible clinical trajectory of combined mucosal immune impairment and opportunistic pathogen co-occurrence. Causality cannot be inferred from a single observation; this case is presented solely to illustrate the biological plausibility of the study hypotheses and to highlight the potential clinical relevance of baseline sIgA in identifying patients at elevated risk of severe oral infectious complications [7,37].

### 4.6. Strengths and Limitations

The principal strengths of this study include its prospective design; the integration of clinical, immunological, microbiological and radiological data within a single cohort; the use of validated composite indices (DMFT/dmft, PUFA/pufa, WHO mucositis grading) that are infrequently applied in paediatric oncology settings; and the use of standardised, non-invasive saliva collection methods appropriate for paediatric patients. To the best of our knowledge, this is one of the first prospective paediatric ALL studies to simultaneously characterise longitudinal salivary sIgA dynamics, OM severity according to WHO grading, and dento-maxillary morbidity burden within an integrated cohort design [9].

The principal limitations of the present study include its single-centre design and limited sample size, partly attributable to the substantial cost of the GI-MAP assay, which reduced statistical power. Salivary data were incomplete, with samples available at diagnosis (TP1) for only 15 of 21 patients and paired TP1–TP3 measurements for only 10 of 21 patients (48%). At TP1, saliva collection was not feasible in six children younger than 4 years because of technical difficulties with non-invasive sampling, whereas at TP3, missing data resulted from the transfer of five patients to other centres following induction chemotherapy and one death during the study period. Because younger children were less likely to provide saliva samples, an age-related selection bias may have influenced the findings and limits their generalisability.

In addition, salivary sIgA was not measured at TP2, corresponding to the period of peak oral mucositis, owing to financial and logistical constraints. Consequently, the temporal relationship between changes in salivary sIgA and the onset, peak severity, or resolution of oral mucositis could not be established, limiting interpretation of salivary sIgA as a dynamic biomarker of mucosal injury.

The absence of a healthy paediatric control group precluded formal comparison of absolute salivary sIgA values against normative references. Missing data are quantified for each analysis by reporting the number of complete observations (n). As this was a convenience sample without an a priori power calculation, the study was underpowered, and post hoc power calculations were not performed because they would not provide additional information.

Methodologically, salivary IgA was quantified by nephelometry (N Latex IgA), which measures total IgA using anti-α-chain antibodies rather than specifically detecting the secretory component. Because salivary IgA is predominantly secretory, the measured concentrations are expected to closely reflect salivary sIgA; however, a secretory component-specific immunoassay was not used. Furthermore, salivary sIgA concentrations are age-dependent in children. As validated age-stratified paediatric reference ranges for this assay were unavailable, absolute concentrations were analysed without age standardisation. Although age was identified as a potential confounder, the limited sample size precluded age-stratified analyses or formal statistical adjustment. Consequently, the observed differences in salivary sIgA concentrations should be interpreted cautiously. Overall, the findings of this exploratory pilot study require confirmation in larger, adequately powered, multicentre prospective cohorts.

## 5. Conclusions

In this exploratory pilot cohort, no statistically significant associations were identified between salivary sIgA and oral mucositis during induction chemotherapy. Although lower baseline salivary sIgA levels in patients who developed more severe oral mucositis and a decline in salivary sIgA following chemotherapy were observed, these findings did not reach statistical significance and should be interpreted cautiously given the limited sample size. Consequently, this study does not establish salivary sIgA as a biomarker of oral mucosal vulnerability in paediatric patients with acute lymphoblastic leukaemia.

The substantial oral and dento-maxillary morbidity documented in this cohort nevertheless supports the systematic integration of dental specialists within the paediatric oncology multidisciplinary team, with an active role in the monitoring and prevention of oral complications throughout treatment. Larger, adequately powered, multicentre prospective studies, with mucositis severity specified as the primary outcome, and including a healthy paediatric control group, are needed to determine whether salivary sIgA has potential as a biomarker of mucosal vulnerability and treatment-related oral toxicity.

## Figures and Tables

**Table 1:** Demographic and disease characteristics of the study cohort (*n* = 21).

Characteristic	Value
Demographics	
Total patients, n	21
Median age, years (IQR; range)	8 (4–12; 2–17)
Male, n (%)	14 (67%)
Female, n (%)	7 (33%)
Urban provenance, n (%)	11 (52%)
Rural provenance, n (%)	8 (38%)
Republic of Moldova, n (%)	2 (10%)
Disease Characteristics	
B-cell ALL, n (%)	16 (76%)
T-cell ALL, n (%)	5 (24%)
Standard-risk group (SRG), n (%)	6 (29%)
Intermediate-risk group, n (%)	11 (52%)
High-risk group (HRG), n (%)	4 (19%)

ALL = acute lymphoblastic leukaemia; IQR = interquartile range; SRG = standard-risk group; HRG = high-risk group. Survival and follow-up status reflect a data cut-off of January 2026; the enrolment window was March–November 2024.

**Table 2:** Salivary sIgA levels at diagnosis (TP1) and after completion of intravenous chemotherapy (TP3).

Parameter	Value
Salivary sIgA at Diagnosis (TP1)	
sIgA TP1 available, n (%)	15/21 (71%)
sIgA TP1—mean ± SD (mg/L)	174.9 ± 152.2
sIgA TP1—median (IQR) (mg/L)	140 (101.5–182)
Salivary sIgA Post-Chemotherapy (TP3)	
sIgA TP3 available, n (%)	15/21 (71%)
sIgA TP3—mean ± SD (mg/L)	122.9 ± 139.1
sIgA TP3—median (IQR) (mg/L)	112 (32.5–125)
Longitudinal Comparison	
Paired TP1–TP3 measurements, n (%)	10 (48%)
TP1 vs. TP3 (Wilcoxon signed-rank test)	*p* = 0.084

sIgA = salivary secretory immunoglobulin A; TP1 = time point 1 (diagnosis); TP3 = time point 3 (post-chemotherapy); SD = standard deviation; IQR = interquartile range.

**Table 3:** Distribution of OM (TP2) severity by ALL immunophenotype and risk group (*n* = 21).

OM (TP2)	ALL Type		Risk Group			Total
	B-Cell	T-Cell	HR	IR	SR	
Grade 1	3	1	1	1	2	4
Grade 2	7	2	2	5	2	9
Grade 3	6	1	0	5	2	7
Grade 4	0	1	1	0	0	1
Total	16	5	4	11	6	21

OM graded according to WHO criteria (grades 0–4). HR = high risk; IR = intermediate risk; SR = standard risk.

**Table 4:** Correlations between dento-maxillary morbidity burden, salivary sIgA and mucositis severity.

	Outcome	rho	*p*	n	*p*_adj	Effect
dmft	sIgA TP1	0.30	0.436	9	0.747	weak
dmft	sIgA TP3	0.53	0.115	10	0.345	moderate
dmft	Oral mucositis	−0.07	0.803	15	0.932	very weak
DMFT	sIgA TP1	0.21	0.531	11	0.796	weak
DMFT	sIgA TP3	−0.83	0.021 *	7	0.162	very strong
DMFT	Oral mucositis	0.03	0.932	11	0.932	very weak
pufa	sIgA TP1	0.44	0.231	9	0.554	moderate
pufa	sIgA TP3	0.55	0.102	10	0.345	moderate
pufa	Oral mucositis	−0.02	0.931	15	0.932	very weak
PUFA	sIgA TP1	0.32	0.334	11	0.668	weak
PUFA	sIgA TP3	−0.81	0.027 *	7	0.162	very strong
PUFA	Oral mucositis	0.10	0.759	11	0.932	very weak

n denotes the number of complete paired observations available for each correlation; it varies across rows because the availability of paired outcome data differed per analysis. * unadjusted *p* < 0.05; no correlation remained significant after Benjamini–Hochberg adjustment.

**Table 5:** Bacteriological Screening Results and Infectious Complications (*n* = 21).

	Negative	Bacteria	Missing
Bacteriological Screening at Diagnosis			
Nose swab	18	1 (*S. aureus* MSSA)	2
Throat swab	15	0	6
Stool sample	18	1 (*E. coli* CARBA+); 1 (*E. faecium* VRE+; *E. coli*)	1
Infectious Complications During Chemotherapy			
Nose swab	15	1 (*S. aureus* MRSA); 1 (*P. aeruginosa* CARBA + MDRO)	4
Throat swab	14	1 (*S. maltophilia*)	6
Stool culture	12	4 (*C. difficile*)	5
Blood culture	14	1 (*P. aeruginosa* MDRO); 1 (CoNS)	5

MSSA = methicillin-susceptible *S. aureus*; MRSA = methicillin-resistant *S. aureus*; CARBA+ = carbapenemase-producing; MDRO = multidrug-resistant organism; VRE+ = vancomycin-resistant *Enterococcus*; CoNS = coagulase-negative *Staphylococcus* spp.

## Data Availability

The data supporting the findings of this study are not publicly available due to privacy and confidentiality restrictions. However, anonymized data may be made available by the corresponding author upon reasonable request.

## Abbreviations

AIEOP-BFM	Associazione Italiana Ematologia Oncologia Pediatrica–Berlin-Frankfurt-Münster
ALL	Acute Lymphoblastic Leukaemia
BCR-ABL1	Breakpoint Cluster Region–Abelson Murine Leukaemia Viral Oncogene Homologue 1 fusion gene
CC BY	Creative Commons Attribution
CI	Confidence Interval
CNS	Central Nervous System
DMFT	Decayed, Missing, Filled Teeth (permanent dentition)
dmft	decayed, missing, filled teeth (primary dentition)
ESCP	European Standard Clinical Practice
ETP-ALL	Early T-cell Precursor Acute Lymphoblastic Leukaemia
ETV6-RUNX1	ETS Variant Transcription Factor 6–RUNX Family Member 1 fusion gene
FAB	French–American–British classification
FDR	False Discovery Rate
FISH	Fluorescence In Situ Hybridisation
GALT	Gut-Associated Lymphoid Tissue
GI-MAP	Gastrointestinal Microbial Assay Plus
HRG	High-Risk Group
iAMP21	Intrachromosomal Amplification of Chromosome 21
IgA	Immunoglobulin A
IQR	Interquartile Range
KMT2A-AFF1	Lysine Methyltransferase 2A–AF4/FMR2 Family Member 1 fusion gene
MALT	Mucosa-Associated Lymphoid Tissue
MDRO	Multidrug-Resistant Organism
MLPA	Multiplex Ligation-dependent Probe Amplification
MRD	Minimal Residual Disease
NF-κB	Nuclear Factor kappa-light-chain-enhancer of activated B cells
NGS	Next-Generation Sequencing
OM	Oral Mucositis
OPG	Orthopantomogram
OS	Overall Survival
PCR	Polymerase Chain Reaction
PUFA	Pulp Involvement, Ulceration, Fistula, Abscess (permanent dentition)
pufa	pulp involvement, ulceration, fistula, abscess (primary dentition)
rpm	Revolutions Per Minute
sIgA	Secretory Immunoglobulin A
SRG	Standard-Risk Group
SROHP	Societatea Română de Onco-Hematologie Pediatrică
STROBE	Strengthening the Reporting of Observational Studies in Epidemiology
TP1	Time Point 1: at diagnosis, prior to chemotherapy initiation
TP2	Time Point 2: peak of induction chemotherapy
TP3	Time Point 3: completion of intravenous chemotherapy
WHO	World Health Organization
